# Matteism—The Latest Craze

**Published:** 1888-11

**Authors:** E. W. Berridge

**Affiliations:** London


### MATTEISM—THE LATEST CRAZE.

E. W. Berridge, M. D., London.

The medical world has just been startled by a new book entitled "*Electro-Homœopathic Medicine:* A New Medical System, being a Popular and Domestic Guide Founded on Experiences by Count Cæsar Mattei. Translated by R. M. Theobald, M. A., M. R. C. S." Had this pretentious work stood merely on its own merits, it might have been dismissed with silent contempt. The Count is not a physician, but simply a well-meaning though very opinionated old gentleman, who has been dabbling in the curative properties of herbs for many years, and thinks he has made a new pathological discovery on which a new and improved system of medicine may be founded. Had he confined his attention to making provings under the guidance of a homœopathic physician so as to insure freedom from ludicrous anatomical blunders, he might have been of some service to humanity. But in posing as a system founder, with the necessary accompaniment of being a system destroyer, he has completely mistaken his *rôle.* His system, which he calls "perfect," and, moreover, has the assurance to call "perfected Homœopathy" and names "Electro-Homœopathy,"* is founded, not on facts, but

---

* Note.—"Electro-Homœopathy" is good. Electro-plate is a spurious imitation of plate; therefore Electro-Homœopathy—need I finish the sentence?

on a pathological theory of his own. And as it happens that this pathological theory is erroneous, the system based thereon must be erroneous also, and all cures made in accordance with it must be accidental. Furthermore, in his attempt to compare his system with that of Hahnemann—of course, to the disparagement of the latter—he makes assertions regarding Hahnemann's teaching which are so wide of the mark, and even in such diametrical opposition to the truth, that the conclusion is irresistibly forced upon one that he never read Hahnemann, but obtained his ideas about Homœopathy from some prejudiced allopath.

But when we find this teaching indorsed by a legally qualified physician, and, moreover, by one whose name appears in the so-called *Homœopathic Directory*, we can no longer ignore it: the matter demands the strictest investigation.

Dr. Theobald says (p. viii): "Those who have carefully experimented with these remedies know that they are capable of exercising extraordinary therapeutic action in many cases which are not amenable to any other known treatment." In illustration of this assertion he declares (p. ix): "My first experiment with Count Mattei's remedies was many years ago, in the case of a young lady reduced to the last extremity of emaciation and exhaustion by marasmus, or wasting disease of the intestines. Ordinary homœopathic treatment by myself, and those who studied the case in consultation with me, produced only unimportant mitigation of symptoms, and in sheer desperation I fell back upon Electro-Homœopathy. Under the use of Scrofoloso and other remedies she rapidly recovered." This is at first sight a remarkable assertion, and one which we cannot ignore. It demands the closest scrutiny. If it be true, then Homœopathy is insufficient, and Electro-Homœopathy must be studied; if it be false, the sooner the bubble is burst the better.

But on attempting to analyze this case we find the necessary elements wanting. Hahnemann's well-known challenge has not been accepted. Had Dr. Theobald published a full account of this case, we should then have been able to ascertain whether the remedies which failed were really homœopathic to the case or not. As it stands at present, it merely shows that certain unknown remedies cured after other unknown remedies had failed. We are none of us infallible, not even the youngest of us, and it is quite possible that Dr. Theobald and his colleagues may have failed to solve the homœopathic equation. This case, therefore, without further details, no more shows the insufficiency of Homœopathy than did the experiments of Andral. I

here repeat the challenge of the master. Will Dr. Theobald take up my gauntlet?

At p. viii Dr. Theobald says, "So far as the relation of these remedies to ordinary Homœopathy is concerned, I regard them myself as homœopathic remedies of a very large range of action." I do not find here that precision of diction which I should have expected from the writer. No remedy can be either homœopathic or allopathic in the abstract. They are homœopathic to a given case only when the symptoms of medicine and patient correspond according to the law of *Similia*, and they are prescribed homœopathically only when administered in accordance with that law. But as Mattei gives no provings of his medicines (indeed, he states that they have no pathogenetic action on the healthy, which is not only a glaring absurdity, but an actual fallacy), and as Dr. Theobald selects them according to Mattei's peculiar pathological theory, and the vague yet complex routine which he indicates, how can he say that they are homœopathic remedies? Possibly he means that their curative action is so pronounced that it must be a homœopathic action, though the prescriber knows it not. If this is his meaning, I agree with him. But why not, then, prove the remedies, that they may be administered with the almost unerring certainty that the law of similars bestows? And if he can find no provers, at least let him give us his cured symptoms. But, perchance, he follows Mattei in his unscientific practice of alternation. This will vitiate every good result which may have been empirically obtained.

Dr. Theobald continues: "Neither the Count nor any of his genuine disciples [alas, poor man! has he also false disciples? If so, he at least in one respect resembles Hahnemann] are disposed to undervalue the transcendent merit of Hahnemann as the greatest medical reformer the world has ever seen." This anticipatory whitewashing of the Count is very ingenious, though I doubt whether he will fully approve of any but himself being declared to be the "greatest medical reformer." Unfortunately for the writer, it is refuted by Mattei's own words. Although Mattei compliments Hahnemann in words (he could hardly do less after having appropriated the name and some of the remedies of Homœopathy), he only places the master on a pinnacle in order that he may soar above him before the eyes of an admiring world. "This people honoreth me with their lips, but their heart is far from me." Neither is the writer more happy in his statement that Matteism is "an extension and simplification of Hahnemannian Homœopathy."

It is rather a limitation and mystification. It, like Schussler's craze, substitutes a limited materia medica for the vast and ever-increasing armamentarium of Homœopathy; it offers us a quasi-allopathic system of polypharmacy for the single remedy of true science, and teaches the selection of the remedy according to pathological fancies (a layman writing on pathology) instead of semiological facts.

Dr. Theobald next gives an additional excuse for preferring Matteism to Hahnemannism: "Those who have endeavored faithfully to apply ordinary Homœopathy according to the directions of Hahnemann, know how prodigiously difficult it is to find a clue to the perplexities of the materia medica, so as to bring its resources within the grasp of persons with an ordinary measure of working capacity and a limited measure of time for attention to individual cases. The real difficulty with ordinary Homœopathy is not in the theory, which is as well demonstrated as any other ascertained law of nature, but in the practice. It seems almost beyond the reach of human faculties to wield such a mighty mass of isolated facts and bring them into actual application. It is too ideal. Its administration requires a *corps* of heaven-born *illuminati*, endowed with an insight or an inspiration not granted to ordinary mortals. It is too esoteric; it postulates a higher order of faculty than that of normal human development. That it will be simplified as it is more diligently worked, I have not the shadow of a doubt; the method of 'keynotes' is a great advance in this direction. Meanwhile, Electro-Homœopathy presents itself as a system in which the rigid individualization of Homœopathy is not required."

Here Dr. Theobald releases from the sack a feline quadruped of remarkable proportions. There's the rub, as Hamlet says. Matteism is valuable—to the lazy and ignorant. But has it never occurred to Dr. Theobald that there can be no royal road to learning, either in Homœopathy or anything else? If a physician is unable to grasp the science and art of Homœopathy, would it not be much better for all concerned that he should turn his attention to some other field than therapeutics, in which he may exercise to the full any other abilities which he may possess? After what Dr. Theobald has written, I almost hesitate to declare that I find none of that "prodigious difficulty" to which he refers; for it might seem like arrogating to myself a sort of divine inspiration. But it is true, nevertheless; and if Dr. Theobald, instead of wasting his undoubted ability on Matteism, had followed my example and striven to complete the repertories up to date, he would not have found himself disappointed.* Dr. Theobald also seems to me to be wide of the mark when he speaks of the keynote method as an "advance" in the simplification of Homœopathy, for it is simply what Hahnemann himself taught when he wrote about characteristic symptoms; but be that as it may, how is Homœopathy to be advanced in the direction of simplification, if her adherents coquette with another mistress? And is not Dr. Theobald's expression, "meanwhile," rather derogatory to Mattei, who claims that "my Electro-Homœopathic system of medicines is the only true and efficient healing force"? I am not at all sure that Matteism is a simplification of treatment. I have carefully studied it, as I do all systems of healing (even the latest idiocy known as Christian Science, on the *lucus a non-lucendo* principle, because there is no Christianity and less science in it, which, so far as it is true, is simply a form of mesmerism, but mixed up with a number of improved theories and arrogant pretensions on the part of some of the "Shrieking Sisterhood"), and I find the rules given of the most indefinite and unsatisfactory character. If it really is simpler than Homœopathy, Matteism may suit some minds just as well as any other form of mongrelism, with the obvious advantage of being both novel and mysterious. But the eulogy here pronounced, that it avoids the "rigid individualization" of Homœopathy, reminds me of a dictionary I once saw, which stated on its title-page that it was "adapted to the meanest capacity." I expected something better than this from one of Dr. Theobald's mental calibre.

Dr. Theobald refers to the "perpetual surprise" which the "magical and marvelous" action of Mattei's remedies causes even "to those who have long used them." Do not the Hahnemannians experience the same? Yea, verily they do; but it is only at the commencement of their investigations; for afterward they are so accustomed to "magical and marvelous" results following the administration of their remedies as surely as effect must ever follow cause, that "surprise" only exists when from some error on their own part these results do not follow.

Dr. Theobald concludes his preface by stating that "there is no real rivalry between Electro-Homœopathy and ordinary Homœopathy;" and that "no one who has noticed the safe, swift, and delightful efficacy of a well-selected† homœopathic remedy can

*Note.—Many years ago I gave Dr. Theobald copies of my MS. repertories, and he acknowledged to me that they had been of immense use to him, leading him to remedies which he could not have otherwise discovered.

†Note.—This admission is a proof that in his quoted case of failure, the remedies were not "well-selected."

consent to forego the advantage of such potent remedial agents. Nor does Count Mattei desire this. He claims for his system that the remedies are illustrative in a conspicuous degree of the homœopathic law." With none of this can I agree. Mattei's system is worse than being the opposite of that of Hahnemann; it is Satan clothed as an angel of light, but, fortunately, only waiting the spear of Ithuriel to unmask the deception. How can he consent to the use of homœopathic adjuncts when he declares, "I have succeeded in discovering a combination of these remedies which may be considered perfect?' If perfect, what need of recourse to aught else? And how can the action of his remedies "illustrate the homœopathic law" till they are proved on healthy persons, which Mattei declares (though in error) is impossible?

Mattei has been in the habit of publishing his indorsement of certain physicians, declaring that he thereby authorizes them to practice Electro-Homœopathy; a piece of arrogant impertinence akin to a telegraph operator giving Edison a testimonial of his proficiency in electricity. Will he continue to indorse Dr. Theobald, who has in this preface flatly contradicted his master and patron by doubting the universality and all-sufficiency of his system?

Our duty as homœopathicians is plain. We must allow no school of medicine, even if founded by a layman, to possess remedies which we cannot scientifically use. It is our duty then to prove these remedies, as we are doing with Schussler's twelve tissue remedies and with the mineral springs which the allopaths sometimes prescribe with great success, though often with serious results.

In the *North American Journal of Homœopathy* for August, 1884, I published what I had discovered respecting these nostrums. The remedies they contain, either singly or in combination, are as follows:

Betonica aquatica.
Brassica oleracea.
Sedum acre.
Sedum telephium.
Sempervivum tectorum.
Matricaria (the wild German chamomile).
Sisymbrium nasturtium.
Thlapsi bursa pastoris.
Chenopodium centinodia.
Persicaria urens (polygonum hydropiper).
Verbena officinalis.

Erysimum officinale.
Gentiana lutea.
Gentiana (grande de chamounix).
Galeopsis grandiflora.

Will the I. H. A. undertake the work?

